# Genetic and Morphological Divergences in the Cosmopolitan Deep-Sea Amphipod *Eurythenes gryllus* Reveal a Diverse Abyss and a Bipolar Species

**DOI:** 10.1371/journal.pone.0074218

**Published:** 2013-09-25

**Authors:** Charlotte Havermans, Gontran Sonet, Cédric d’Udekem d’Acoz, Zoltán T. Nagy, Patrick Martin, Saskia Brix, Torben Riehl, Shobhit Agrawal, Christoph Held

**Affiliations:** 1 Direction Natural Environment, Royal Belgian Institute of Natural Sciences, Brussels, Belgium; 2 Direction Taxonomy and Phylogeny, Royal Belgian Institute of Natural Sciences, Brussels, Belgium; 3 Biodiversity Research Centre, Earth and Life Institute, Catholic University of Louvain, Louvain-la-Neuve, Belgium; 4 Centre for Marine Biodiversity Research, Senckenberg Research Institute c/o Biocentrum Grindel, Hamburg, Germany; 5 Section Functional Ecology, Alfred Wegener Institute Helmholtz Centre for Polar and Marine Research, Bremerhaven, Germany; Consiglio Nazionale delle Ricerche (CNR), Italy

## Abstract

*Eurythenes gryllus* is one of the most widespread amphipod species, occurring in every ocean with a depth range covering the bathyal, abyssal and hadal zones. Previous studies, however, indicated the existence of several genetically and morphologically divergent lineages, questioning the assumption of its cosmopolitan and eurybathic distribution. For the first time, its genetic diversity was explored at the global scale (Arctic, Atlantic, Pacific and Southern oceans) by analyzing nuclear (28S rDNA) and mitochondrial (COI, 16S rDNA) sequence data using various species delimitation methods in a phylogeographic context. Nine putative species-level clades were identified within *E. gryllus*. A clear distinction was observed between samples collected at bathyal versus abyssal depths, with a genetic break occurring around 3,000 m. Two bathyal and two abyssal lineages showed a widespread distribution, while five other abyssal lineages each seemed to be restricted to a single ocean basin. The observed higher diversity in the abyss compared to the bathyal zone stands in contrast to the depth-differentiation hypothesis. Our results indicate that, despite the more uniform environment of the abyss and its presumed lack of obvious isolating barriers, abyssal populations might be more likely to show population differentiation and undergo speciation events than previously assumed. Potential factors influencing species’ origins and distributions, such as hydrostatic pressure, are discussed. In addition, morphological findings coincided with the molecular clades. Of all specimens available for examination, those of the bipolar bathyal clade seemed the most similar to the ‘true’ *E. gryllus*. We present the first molecular evidence for a bipolar distribution in a macro-benthic deep-sea organism.

## Introduction

The deep sea represents the largest ecosystem on our planet, yet our awareness of its fauna is remarkably recent, dating back to the Challenger expedition (1872–1876) (e.g. [1]). In the last decades, an increasing number of studies were conducted to investigate the deep-sea benthic biodiversity in several regions of the world, revealing its unexpectedly high species diversity and endemism [2], [3], [4], [5]. Yet, little is known about how this remarkable fauna has evolved. The supposed lack of isolating barriers in the deep sea and the global uniformity of its environment contributed to the belief in cosmopolitan distributions of taxa (e.g. [6]). Previously observed bathymetric patterns in diversity suggest a maximal diversity at bathyal depths, decreasing towards the abyss [7], [8]. Indeed, the bathyal region is characterized by stronger abiotic and biotic gradients and greater habitat heterogeneity in comparison to the abyss. Hence, population differentiation, and ultimately, speciation events, are supposed to be more common in the bathyal zone (i.e. the depth-differentiation hypothesis, reviewed in [9]).

Many deep-sea species have geographic distributions encompassing one or more entire oceans as well as very wide bathymetric ranges (e.g. [10]). The assessment of population structure and diversity using molecular methods has been applied to deep-sea fauna only in the last two decades. First insights into genetic variation of deep-sea organisms suggest that many species, reported to be widespread based on morphological criteria, may comprise multiple cryptic species [11], [12], [13], [14], [15], [16], [17]. These findings challenge the hypothesis of a cosmopolitan deep-sea fauna and the validity of these species should therefore be further tested with modern molecular methods.

An example of such presumably cosmopolitan species is *Eurythenes gryllus* (Lichtenstein, 1822), a giant bentho-pelagic deep-sea amphipod. Among all known lysianassoid species, *E. gryllus* has the widest geographical distribution. It occurs in every ocean and has a depth range from 550 to 7,800 m, covering the bathyal, abyssal and hadal zones [10], [18], [19], [20], [21]. With its specialized necrophagous feeding mode, it has an important role in the deep-sea benthic food web by consuming large particles that fall to the ocean floor. This scavenger is perhaps the most remarkable bait-attending amphipod, appearing rapidly and in high numbers at carcasses or baited traps, due to its long-range chemoreceptive tracking ability [22] and considerable swimming speed [23]. Its biology has been extensively studied in many aspects: feeding strategy and meal sizes (e.g. [24], [25], [26], [27]), metabolism and respiration rate (e.g. [23], [28]), pigment physiology [29], population biology and vertical distribution (e.g. [20], [30], [31], [32], [33]).

However, *E. gryllus* was found to comprise several morphologically distinct and genetically divergent lineages, which seem to be vertically stratified [12], [18], [19], [34], [35]. Barnard [18] noted differences of gnathopod structure between populations of small and large individuals. Size appeared to differ between non-abyssal and abyssal individuals, with the former being smaller [12]. This vertical separation was also confirmed by molecular evidence. It was firstly highlighted with allozymic data, showing a significant differentiation between seamount slope and abyssal plain specimens [34]. In contrast, comparatively little horizontal differentiation was observed between populations that are spatially separated by 4,000 km in the Pacific Ocean [34]. The relatively larger-scale analysis of France and Kocher [12] obtained similar patterns based on mitochondrial 16S rDNA sequence diversity, i.e. a much higher genetic divergence over the bathymetric range compared to the geographic scale. They discovered five differentiated lineages in the bathyal zone while abyssal specimens were genetically similar. Recently, 16S rDNA sequence analyses revealed genetic homogeneity between specimens from two abyssal locations in the Gulf of Mexico but considerable genetic divergence between these populations and those in the western Atlantic bathyal zone [36]. Finally, isolation of a hadal population in a deep-sea trench was suggested based on morphological differences exceeding the known intraspecific variability [20]. These results suggest that extending molecular and morphological studies to other geographic and bathymetric ranges will reveal even more lineages or overlooked species.

Aforementioned studies were generally carried out on a limited geographic scale and solely based on either morphology or genetic analyses using a single molecular marker. A study at a worldwide scale, based on a combined approach by both molecular and morphological tools has not yet been undertaken. Therefore, we explored genetic diversity in *E. gryllus* at the global scale (Arctic, Atlantic, Pacific and Southern oceans) using both nuclear and mitochondrial DNA sequence data. We tested for evidence of multiple species and we attempted to identify potential barriers in the deep sea that could isolate gene pools and allow populations to diverge into separate species. Finally, an analysis of morphological characters was performed to detect the possible existence of previously overlooked morphological differences within *E. gryllus* and to ascertain which of the genetic lineages, if any, is identical to the material described as *E. gryllus*.

## Materials and Methods

### Sampling of New Material and Morphological Analysis

**Figure 1:**
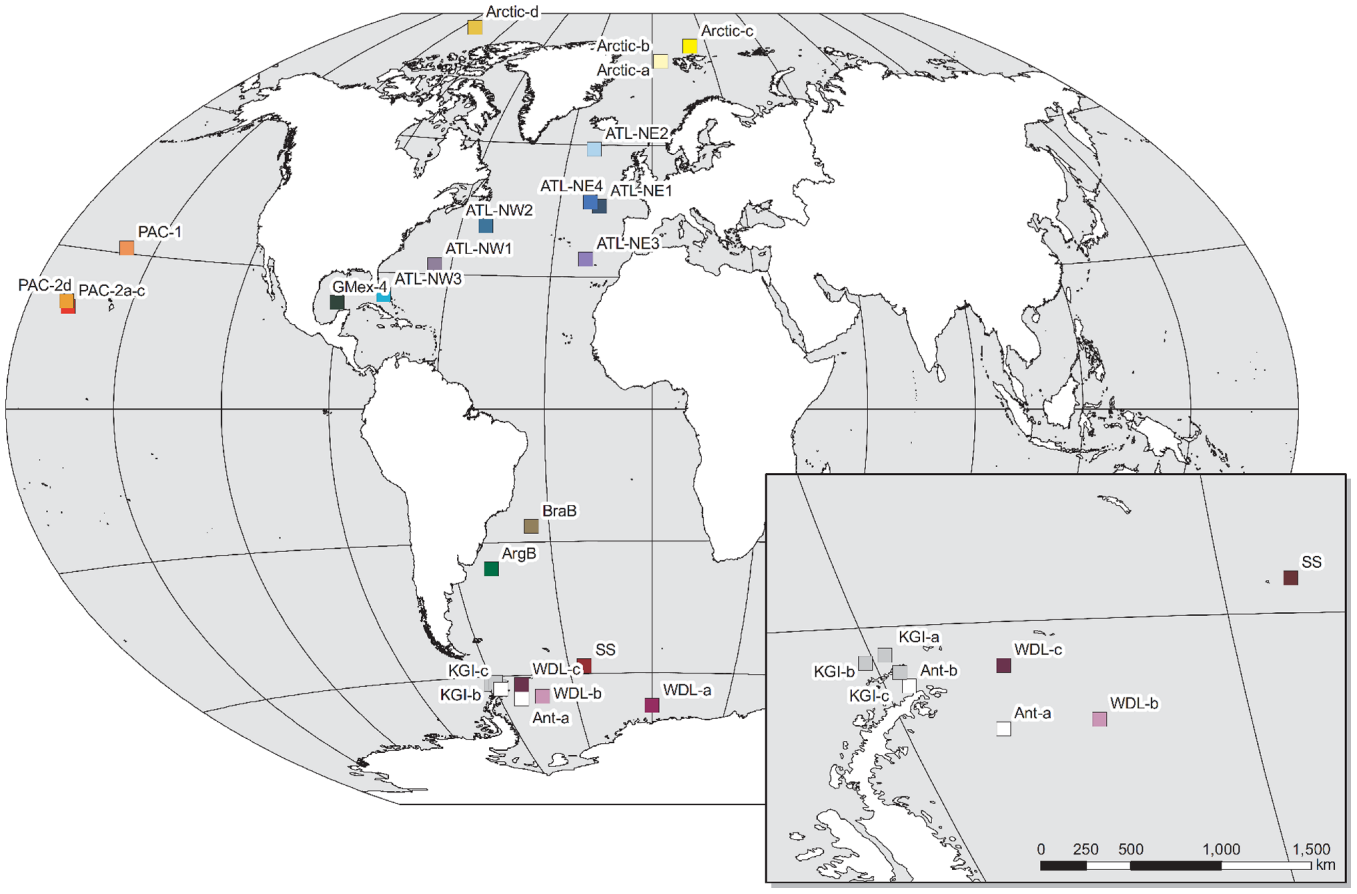
Sample localities of *Eurythenes gryllus sensu lato*. Abbreviations refer to samples from this study, that of France and Kocher [12] and Escobar-Briones *et al.* [36] (for details see Table 1). The sampling region in the Southern Ocean is shown as an enlargement. Color codes are provided for each sampling locality and are used consistently in the other figures.

Specimens of *E. gryllus* were collected during recent expeditions of *RV Polarstern* and *RV Meteor*. Traps were deployed at the following locations: the Arctic Ocean, Brazil and Argentine Basins and several locations in the Southern Ocean. Permission to collect amphipod samples from the prospected areas in the Southern Ocean was obtained from the Belgian authorities observing the guidelines of the Environment Protocol to the Antarctic Treaty (Permit nr. 01/10). None of the other sampling stations were within specially protected areas and no permits were required for work in these locations. *Eurythenes gryllus* is not an endangered or protected species. Sampling details are listed in Table 1. Depths from 839 to 4,693 m were sampled, comprising the bathyal zone and the abyss (here defined by depths between 3,000 and 6,000 m, according to Smith *et al.* [37]). The amphipods were fixed in 96–100% ethanol. These stations, together with the stations sampled by France and Kocher [12] and Escobar-Briones *et al.* [36], from which sequence data were included in our analyses, are represented on Figure 1. A morphological examination was carried out on the newly collected specimens. Specimens were measured for total length (from the rostrum to the tip of the telson) and whenever possible, sex was determined. Complete specimens and appendages were examined with a particular attention to characters used in lysianassoid taxonomy and were compared with previous morphological studies [18], [19], [20], [21], [38] as well as with the holotype of *E. gryllus*.

**Table 1:** Data of specimens of *Eurythenes gryllus sensu lato* (EG) and *Eurythenes* sp. (ES) obtained for this study and available on GenBank from France and Kocher [12] and Escobar-Briones *et al.* [36].

Abbreviation	IdentificationCode	Samplinglocation	Expedition	Station	Coordinates	Depth(m)	Accession numbers		
							COI	28S	16S
**This study**									
KGI-a1	EG-0112106	North of King George Island	ANDEEP I & II	100 BT	61°25′S 58°53′W	2280	JX887134	JX887087	n.d.
KGI-b1	EG-0112107	North of King George Island	ANDEEP I & II	114 BT	61°45′S 60°45′W	2743	JX887135	JX887087	JX887062
Ant-a1	EG-0112105	East of Antarctic Peninsula	ANDEEP I & II	131-1 BT	65°17′S 51°35′W	3070	JX887112	JX887078	JX887065
Ant-a2	EG-0112104	East of Antarctic Peninsula	ANDEEP I & II	131-1 BT	65°17′S 51°35′W	3070	JX887116	JX887077	JX887065
Ant-a3	EG-0112102	East of Antarctic Peninsula	ANDEEP I & II	131-1 BT	65°17′S 51°35′W	3070	JX887123	JX887103	JX887067
Ant-a4	EG-P3049	East of Antarctic Peninsula	ANDEEP I & II	131-1 BT	65°17′S 51°35′W	3070	GU109270	JX887104	JX887067
SS-1	EG-0112103	South Sandwich Islands	ANDEEP I & II	139-1 BT	58°17′S 24°29′W	3739	JX887120	JX887076	JX887065
SS-2	EG-28071120	South Sandwich Islands	ANDEEP I & II	139-1 BT	58°17′S 24°29′W	3739	JX887116	JX887078	JX887065
SS-3	EG-28071121	South Sandwich Islands	ANDEEP I & II	139-1 BT	58°17′S 24°29′W	3739	n.d.	n.d.	JX887065
SS-4	EG-28071123	South Sandwich Islands	ANDEEP I & II	139-1 BT	58°17′S 24°29′W	3739	n.d.	n.d.	JX887065
WDL-a1	EG-21101012	Eastern Weddell Sea	ANDEEP III	59 BT	67°30′S 00°00′W	4625	JX887114	JX887079	JX887065
WDL-a2	EG-0112101	Eastern Weddell Sea	ANDEEP III	59 BT	67°30′S 00°00′W	4625	JX887138	JX887075	JX887065
WDL-a3	EG-28071124	Eastern Weddell Sea	ANDEEP III	59 BT	67°30′S 00°00′W	4625	n.d.	n.d.	JX887065
WDL-b1	EG-28071113	Weddell Sea	ANDEEP III	110 BT	64°56′S 43°08′W	4693	JX887116	JX887081	JX887065
WDL-b2	EG-28071114	Weddell Sea	ANDEEP III	110 BT	64°56′S 43°08′W	4693	JX887119	n.d.	JX887066
WDL-b3	EG-28071115	Weddell Sea	ANDEEP III	110 BT	64°56′S 43°08′W	4693	JX887116	JX887081	JX887065
WDL-b4	EG-28071116	Weddell Sea	ANDEEP III	110 BT	64°56′S 43°08′W	4693	JX887115	JX887078	JX887065
WDL-b5	EG-28071117	Weddell Sea	ANDEEP III	110 BT	64°56′S 43°08′W	4693	n.d.	n.d.	JX887065
WDL-b6	EG-28071118	Weddell Sea	ANDEEP III	110 BT	64°56′S 43°08′W	4693	JX887116	JX887082	JX887065
WDL-b7	EG-28071119	Weddell Sea	ANDEEP III	110 BT	64°56′S 43°08′W	4693	n.d.	n.d.	JX887065
WDL-c1	EG-2807118	Weddell Sea - Scotia Sea	ANDEEP III	142 BT	62°12′S 49°28′W	3407	JX887117	n.d.	JX887065
WDL-c2	EG-2807119	Weddell Sea - Scotia Sea	ANDEEP III	142 BT	62°12′S 49°28′W	3407	JX887113	n.d.	JX887065
WDL-c3	EG-28071110	Weddell Sea - Scotia Sea	ANDEEP III	142 BT	62°12′S 49°28′W	3407	JX887116	JX887080	JX887065
WDL-c4	EG-28071111	Weddell Sea - Scotia Sea	ANDEEP III	142 BT	62°12′S 49°28′W	3407	JX887118	JX887078	JX887065
WDL-c5	EG-28071112	Weddell Sea - Scotia Sea	ANDEEP III	142 BT	62°12′S 49°28′W	3407	n.d.	n.d.	JX887065
Ant-b1	EG-1412101	North of Antarctic Peninsula	ANT XXIII-8	683-1FT	62°58′S 57°58′W	839	JX887140	JX887088	JX887060
Ant-b2	EG-1412102	North of Antarctic Peninsula	ANT XXIII-8	683-1FT	62°58′S 57°58′W	839	JX887139	JX887089	JX887060
KGI-c1	EG-ANT273-20	King George Island	ANT XXVII-3	223-1 FT	62°17′S 58°17′W	980	JX887142	JX887090	JX887060
KGI-c2	EG-ANT273-21	King George Island	ANT XXVII-3	223-1 FT	62°17′S 58°17′W	980	JX887141	JX887091	JX887060
KGI-c3	EG-ANT273-22	King George Island	ANT XXVII-3	223-1 FT	62°17′S 58°17′W	980	JX887133	JX887092	JX887060
KGI-c4	EG-ANT273-23	King George Island	ANT XXVII-3	223-1 FT	62°17′S 58°17′W	980	JX887136	JX887092	JX887060
KGI-c5	EG-ANT273-24	King George Island	ANT XXVII-3	223-1 FT	62°17′S 58°17′W	980	JX887136	JX887093	JX887060
KGI-c6	EG-ANT273-45	King George Island	ANT XXVII-3	223-1 FT	62°17′S 58°17′W	980	JX887136	JX887094	JX887060
KGI-c7	EG-ANT273-46	King George Island	ANT XXVII-3	223-1 FT	62°17′S 58°17′W	980	JX887136	n.d.	JX887060
ArgB-1	EG-1102101	Argentine Basin	M79/1 DIVA 3	531 BT	35°56′S 48°54′W	4586	JX887121	JX887105	JX887067
ArgB-2	EG-1102102	Argentine Basin	M79/1 DIVA 3	531 BT	35°56′S 48°54′W	4586	JX887137	JX887106	JX887067
ArgB-3	EG-1102103	Argentine Basin	M79/1 DIVA 3	531 BT	35°56′S 48°54′W	4586	JX887125	n.d.	JX887068
ArgB-4	EG-2110109	Argentine Basin	M79/1 DIVA 3	531 BT	35°56′S 48°54′W	4586	JX887124	JX887108	JX887067
ArgB-5	EG-21101010	Argentine Basin	M79/1 DIVA 3	531 BT	35°56′S 48°54′W	4586	JX887122	JX887107	JX887068
ArgB-6	EG-21101011	Argentine Basin	M79/1 DIVA 3	531 BT	35°56′S 48°54′W	4586	n.d.	n.d.	JX887069
ArgB-7	EG-1810111	Argentine Basin	M79/1 DIVA 3	531 BT	35°56′S 48°54′W	4586	JX887151	n.d.	JX887069
ArgB-8	EG-1810112	Argentine Basin	M79/1 DIVA 3	531 BT	35°56′S 48°54′W	4586	JX887152	JX887111	JX887069
ArgB-9	EG-1810113	Argentine Basin	M79/1 DIVA 3	531 BT	35°56′S 48°54′W	4586	JX887151	JX887110	JX887069
ArgB-10	EG-1810114	Argentine Basin	M79/1 DIVA 3	531 BT	35°56′S 48°54′W	4586	JX887151	n.d.	JX887069
BraB-1	Euryt 77412540	Brazil Basin	M79/1 DIVA 3	542 BT	26°33′S 35°11′W	4480	JX887144	JX887101	JX887071
BraB-2	Euryt 77412554	Brazil Basin	M79/1 DIVA 3	542 BT	26°33′S 35°11′W	4480	JX887144	JX887102	JX887072
BraB-3	Euryt 77412514	Brazil Basin	M79/1 DIVA 3	542 BT	26°33′S 35°11′W	4480	JX887143	JX887100	JX887071
BraB-4	Euryt 77412506	Brazil Basin	M79/1 DIVA 3	542 BT	26°33′S 35°11′W	4480	JX887145	JX887097	JX887073
BraB-5	Euryt 77412578	Brazil Basin	M79/1 DIVA 3	542 BT	26°33′S 35°11′W	4480	JX887145	JX887099	JX887074
BraB-6	Euryt 77412562	Brazil Basin	M79/1 DIVA 3	542 BT	26°33′S 35°11′W	4480	JX887145	JX887098	JX887073
BraB-7	Euryt 77412564	Brazil Basin	M79/1 DIVA 3	542 BT	26°33′S 35°11′W	4480	JX887146	n.d.	JX887073
BraB-8	EG-2101108	Brazil Basin	M79/1 DIVA 3	542 BT	26°33′S 35°11′W	4480	n.d.	n.d.	JX887070
Arctic-a1	EG-0112108	Eastern Fram Strait	ARK XIX-3	412-1 BT	79°04′N 04°08′E	2464	JX887129	JX887087	JX887060
Arctic-a2	EG-01121012	Eastern Fram Strait	ARK XIX-3	412-1 BT	79°04′N 04°08′E	2464	JX887132	JX887084	JX887060
Arctic-a3	EG-01121013	Eastern Fram Strait	ARK XIX-3	412-1 BT	79°04′N 04°08′E	2464	JX887127	JX887085	JX887064
Arctic-a4	EG-01121014	Eastern Fram Strait	ARK XIX-3	412-1 BT	79°04′N 04°08′E	2464	JX887128	JX887086	JX887063
Arctic-a5	EG-01121015	Eastern Fram Strait	ARK XIX-3	412-1 BT	79°04′N 04°08′E	2464	JX887130	JX887087	JX887060
Arctic-a6	EG-01121016	Eastern Fram Strait	ARK XIX-3	412-1 BT	79°04′N 04°08′E	2464	JX887126	JX887084	JX887060
Arctic-b1	EG-0112109	Eastern Fram Strait	ARK XIX-3	423-1 BT	79°03′N 04°16′E	2461	JX887131	JX887084	JX887060
Arctic-b2	EG-01121010	Eastern Fram Strait	ARK XIX-3	423-1 BT	79°03′N 04°16′E	2461	JX887132	JX887083	JX887060
Arctic-c1	EG-1810115	Svalbard archipelago	Jan Mayen 2004	n.d.	82°26′N 20°52′E	1660	JX887148	JX887095	JX887060
Arctic-c2	EG-1810116	Svalbard archipelago	Jan Mayen 2004	n.d.	82°26′N 20°52′E	1660	JX887147	n.d.	JX887060
Arctic-c3	EG-1810119	Svalbard archipelago	Jan Mayen 2004	n.d.	82°26′N 20°52′E	1660	JX887147	n.d.	JX887061
Arctic-c4	EG-18101110	Svalbard archipelago	Jan Mayen 2004	n.d.	82°26′N 20°52′E	1660	JX887147	JX887109	JX887060
Arctic-c5	EG-18101111	Svalbard archipelago	Jan Mayen 2004	n.d.	82°26′N 20°52′E	1660	JX887149	n.d.	JX887060
Arctic-c6	EG-18101112	Svalbard archipelago	Jan Mayen 2004	n.d.	82°26′N 20°52′E	1660	JX887147	JX887109	JX887060
Arctic-c7	EG-18101113	Svalbard archipelago	Jan Mayen 2004	n.d.	82°26′N 20°52′E	1660	JX887150	JX887096	JX887060
**France and Kocher ** [**12**]									
Arctic-d (4)	EG-U40437	Canada Basin	n.d.	n.d.	86°N 111°W	2076	n.d.	n.d.	U40437
ATL-NE1 (10)	EG-U40438	Iberia Abyssal Plain	n.d.	n.d.	46°N 17°W	4695	n.d.	n.d.	U40438
ATL-NE2 (5)	EG-U40439	Iceland Basin	n.d.	n.d.	59°N 21°W	2900	n.d.	n.d.	U40439
ATL-NE2 (1)	EG-U40440	Iceland Basin	n.d.	n.d.	59°N 21°W	2900	n.d.	n.d.	U40440
ATL-NE3 (6)	EG-U40438	Madeira Abyssal Plain	n.d.	n.d.	34°N 20°W	5117	n.d.	n.d.	U40438
ATL-NE4 (9)	EG-U40438	West European Basin	n.d.	n.d.	47°N 20°W	3860–4570	n.d.	n.d.	U40438
ATL-NW1 (4)	EG-U40438	Nares Abyssal Plain	n.d.	n.d.	32°N 65°W	3526	n.d.	n.d.	U40438
ATL-NW2 (9)	EG-U40438	Sohm Abyssal Plain	n.d.	n.d.	41°N 52°W	4983	n.d.	n.d.	U40438
ATL-NW3a (10)	EG-U40440	Tongue of Ocean (TOTO), Bahamas	n.d.	n.d.	25°N 78°W	1309	n.d.	n.d.	U40440
PAC-1 (7)	EG-U40441	Central North Pacific	n.d.	n.d.	31°N 159°W	5770	n.d.	n.d.	U40441
PAC-1 (7)	EG-U40442	Central North Pacific	n.d.	n.d.	31°N 159°W	5770	n.d.	n.d.	U40442
PAC-2a (1)	EG-U40441	Horizon Guyot base	n.d.	n.d.	19°N 168°W	5178	n.d.	n.d.	U40441
PAC-2a (1)	EG-U40442	Horizon Guyot base	n.d.	n.d.	19°N 168°W	5178	n.d.	n.d.	U40442
PAC-2a (1)	EG-U40443	Horizon Guyot base	n.d.	n.d.	19°N 168°W	5178	n.d.	n.d.	U40443
PAC-2b (3)	EG-U40441	Horizon Guyot base	n.d.	n.d.	20°N 169°W	4920	n.d.	n.d.	U40441
PAC-2b (7)	EG-U40442	Horizon Guyot base	n.d.	n.d.	20°N 169°W	4920	n.d.	n.d.	U40442
PAC-2b (2)	EG-U40444	Horizon Guyot base	n.d.	n.d.	20°N 169°W	4920	n.d.	n.d.	U40444
PAC-2c (1)	EG-U40441	Horizon Guyot slope	n.d.	n.d.	20°N 169°W	3982	n.d.	n.d.	U40441
PAC-2c (1)	EG-U40442	Horizon Guyot slope	n.d.	n.d.	20°N 169°W	3982	n.d.	n.d.	U40442
PAC-2c (1)	EG-U40445	Horizon Guyot slope	n.d.	n.d.	20°N 169°W	3982	n.d.	n.d.	U40445
PAC-2d (1)	EG-U40446	Horizon Guyot slope	n.d.	n.d.	20°N 169°W	3193	n.d.	n.d.	U40446
PAC-2d (4)	EG-U40447	Horizon Guyot slope	n.d.	n.d.	20°N 169°W	3193	n.d.	n.d.	U40447
PAC-2d (1)	EG-U40448	Horizon Guyot slope	n.d.	n.d.	20°N 169°W	3193	n.d.	n.d.	U40448
ATL-NW3b (3)	ES-U40449	NW Channel, Bahamas	n.d.	n.d.	25°N 78°W	1122	n.d.	n.d.	U40449
**Escobar-Briones ** ***et al.*** [**36**]									
GMex-4	EG-AY943568	Gulf of Mexico	n.d.	St 2	23°N 91°W	3732	n.d.	n.d.	AY943568

For the specimens sequenced by France and Kocher [12], the number of specimens is added between parentheses. Abbreviations: n.d. – no data, BT – baited traps, FT – fish traps.

### Laboratory Techniques

Genomic DNA was isolated from pereopod 6 using the NucleoSpin Tissue kit (Macherey-Nagel). PCR amplifications of the mitochondrial COI and 16S rRNA gene fragments were carried out using the universal primers LCO1490, HCO2198 [39] and 16Sar, 16Sbr [40], respectively. Amplification of a fragment of the nuclear marker 28S rDNA was performed using the primers 28F and 28R [41]. PCR settings for amplifying COI and 28S are described in detail in the study of Havermans *et al.* [42], and those for 16S in the study of France and Kocher [12]. Purified PCR products were sequenced bi-directionally using an ABI 3130×l capillary DNA sequencer (Life Technologies). New sequences were deposited in GenBank (Acc. Nos. JX887060–JX887152).

### Species Delimitation and Phylogeographic Analyses

Chromatograms were checked and sequence assembly was carried out with CodonCode Aligner v2.0.6 (Codon Code Corporation). COI sequences were aligned manually; 16S and 28S rDNA sequences with the MAFFT 6 web server (using the G-INS-i option) [43], [44]. In order to prevent inclusion of pseudogenes in the analyses, electropherograms were checked for ambiguous base calls and amino acid translations of the COI sequences for stop codons.

Tree-construction methods were used to identify possible clades within *E. gryllus*, using the combined dataset and 16S rDNA dataset. The combined dataset consisted of COI, 16S and 28S rDNA sequences of 47 specimens collected for this study. Identical sequences were collapsed into unique haplotypes, resulting in 45 concatenated sequences, which were used to perform parsimony and Bayesian analyses. Sequences of the lysianassoid *Abyssorchomene* spp., available from GenBank, were used to define the outgroup (*A.* sp.: 16S Acc. No. U40450; *A. chevreuxi*: 28S Acc. No. GU109197, COI Acc. No. GU109229). The 16S rDNA dataset generated in this study (66 sequences) was complemented by sequences of *E. gryllus* available in GenBank: 96 sequences representing 12 different haplotypes from France and Kocher [12] and one sequence from Escobar-Briones *et al.* [36] (details in Table 1). This dataset consisted of 27 unique haplotypes. Three specimens of *Eurythenes* sp. (U40449) were recognized as a distinct species by France and Kocher [12], [35] and treated as *E. thurstoni* in this study (according to Stoddart and Lowry [21]). Parsimony analyses were performed on the combined dataset using PAUP* 4.0b10 [45], with all characters equally weighted and unordered. Alignment gaps were treated as a fifth character or alternatively as missing data. Heuristic searches were carried out with random sequence addition (10 replicates) and using tree-bisection-reconnection (TBR) branch swapping. Branch support was inferred using non-parametric bootstrapping, with 2,000 replicates. Bayesian analyses were performed both on the combined and the 16S dataset. For the former, five data partitions were used: three partitions for each codon position of COI and one partition for 16S and 28S each. The best-fit substitution models were selected using jModeltest 0.1.1 [46] by estimating and comparing maximum likelihood scores for different nucleotide substitution models and this was done for each of the data partitions. The Bayesian Information Criterion (BIC; [47]) was used to identify the best-fit models: TPM1+I+G for position 1 of COI, F81 for position 2 of COI, TPM3+G for position 3 of COI, HKY+G for 16S and TPM1+I+G for 28S. These models were used in Bayesian analyses performed by MrBayes 3.1.2 [48]. Two parallel runs with four chains each were run for 10 million generations, every 1,000th generation was sampled (resulting in 10,000 trees). Convergence of runs was monitored using Tracer v1.5 and the first 50% of the trees were discarded as burn-in, while the last 5,000 trees were used to reconstruct a consensus tree and estimate Bayesian posterior probabilities.

Relationships between haplotypes were investigated by generating haplotype networks with the complete 16S dataset (163 sequences) using TCS 1.21 [49], with gaps considered as a fifth state and a 95% probability threshold. Additional sequences of COI and 16S of *E. gryllus* from GenBank [35] were not included in this study, since most of these sequences were recovered from formalin-fixed individuals and thus very short (around 200 bp).

Single gene datasets of the three genes were used for identifying species complexes from genetic divergence between specimens (distance-based approach *sensu* Hebert *et al.* [50]). Since divergences within species are generally smaller than divergences among species, we searched for the presence of a barcoding gap in the distribution of all pairwise distances, i.e. an interval between the highest intraspecific and the lowest interspecific distances. Divergences were compared for all three genes in order to delimit species-level clades based on a distance threshold. Sequence divergences were calculated using the Kimura 2-parameter (K2P) distance model [51] on the COI, 16S and 28S dataset of 59, 66 and 48 sequences, respectively, with MEGA 5 [52]. Although the use of this K2P correction has been discussed, the accuracy of distance-based identifications does not differ significantly when using p-distances (e.g. [53]). Further, it allows comparison with previous studies on lysianassoids [54].

Finally, the method of Pons *et al.* [55] was used to identify independent lineages with the 16S rDNA dataset. This likelihood method determines the point of transition from speciation to coalescent branching patterns on an ultrametric tree. It uses the predicted difference in branching rates between and within species, identifying the point where a transition is the most likely compared with a null model that all sequences are derived from a single species [55], [56]. We used both single-threshold and multiple-threshold general mixed Yule-coalescent (GMYC) models as implemented in the R package ‘splits’ for the ultrametric trees obtained by Bayesian analyses to test the existence of several species within *E. gryllus*.

## Results

### Species Delimitation and Phylogeographic Analyses

The three-gene dataset comprised 45 unique concatenated sequences, each consisting of 2421 positions. The aligned COI sequences consisted of 658 bases, of which 119 were parsimony-informative. Translation revealed a higher mutation rate at third codon positions, no ambiguous base calls and no stop codons, which is typical for functional protein-coding genes as opposed to pseudogenes. The aligned 16S and 28S rDNA sequences contained 489 and 1274 positions of which 123 and 11 were parsimony-informative, respectively.

**Figure 2:**
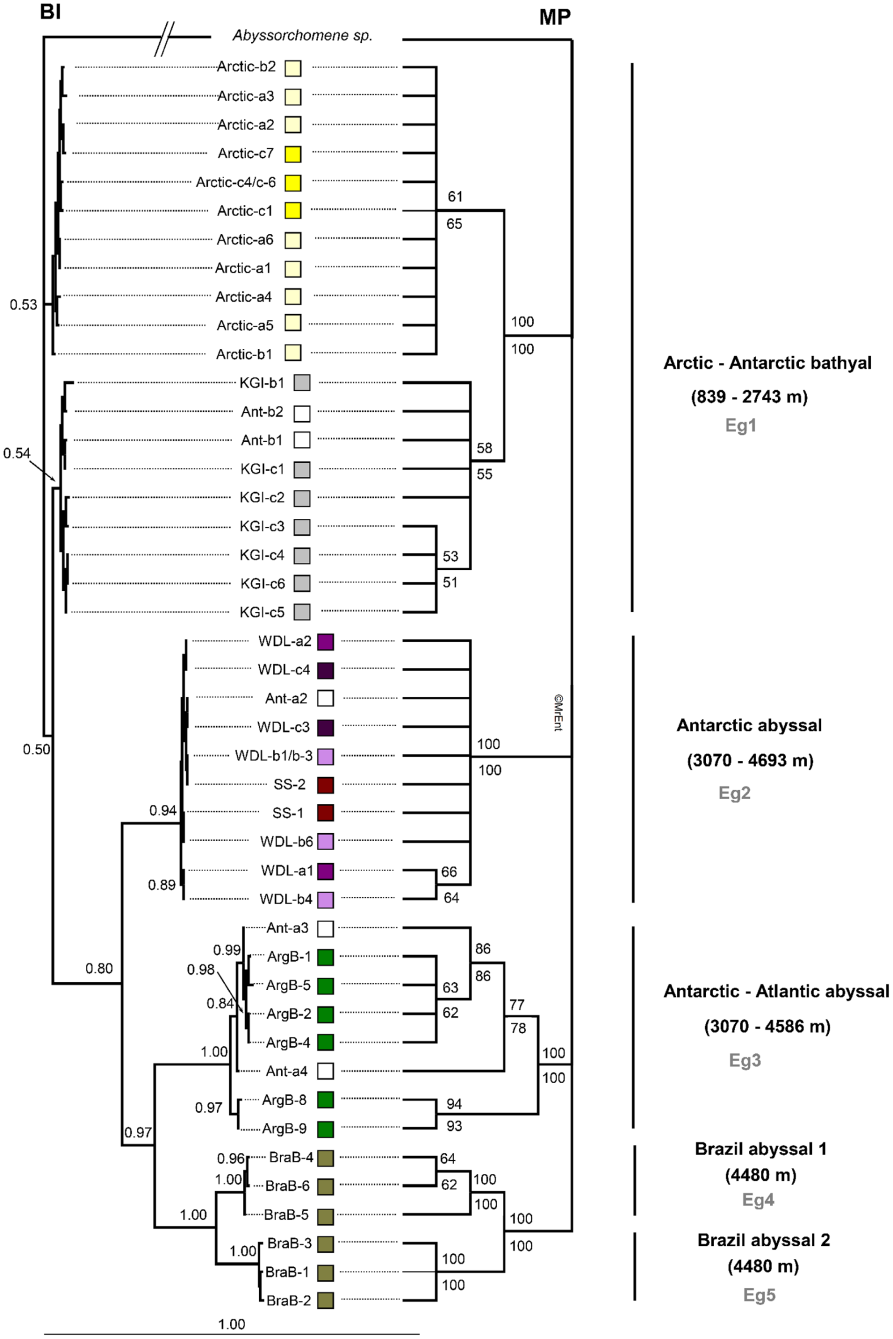
Tree constructions on the three-gene dataset. Bayesian (BI) and Maximum Parsimony (MP) trees inferred for specimens of *Eurythenes gryllus* sampled in this study, based on the combined dataset of three genes (COI, 16S rDNA, 28S rDNA), showing posterior probabilities (>0.5) and bootstrap values (>50%; number of bootstrap replicates = 2,000), respectively. Two bootstrap values are shown at each node, the upper one represents the value when gaps were treated as fifth characters whilst the lower one represents the value when gaps were treated as missing data. The different clusters are assigned with the codes Eg1–5. For each cluster, distributional ranges (ocean basin, bathyal vs. abyssal, depth) are indicated. The colored squares refer to the sample localities of Figure 1.

The Bayesian and parsimony reconstructions of the combined dataset are represented in Figure 2. In the parsimony tree, the clade comprising both Antarctic and Arctic bathyal specimens (Eg1) received a high (100%) bootstrap support. This clade was unresolved in the Bayesian tree. Four other strongly supported clades could be distinguished in both the Bayesian and parsimony trees: Eg2, comprising Antarctic abyssal (>3,000 m) specimens; Eg3, comprising Antarctic and Atlantic abyssal (Argentine abyssal Basin) specimens; Eg4 and Eg5, both comprising specimens from the Brazil abyssal Basin.

**Figure 3:**
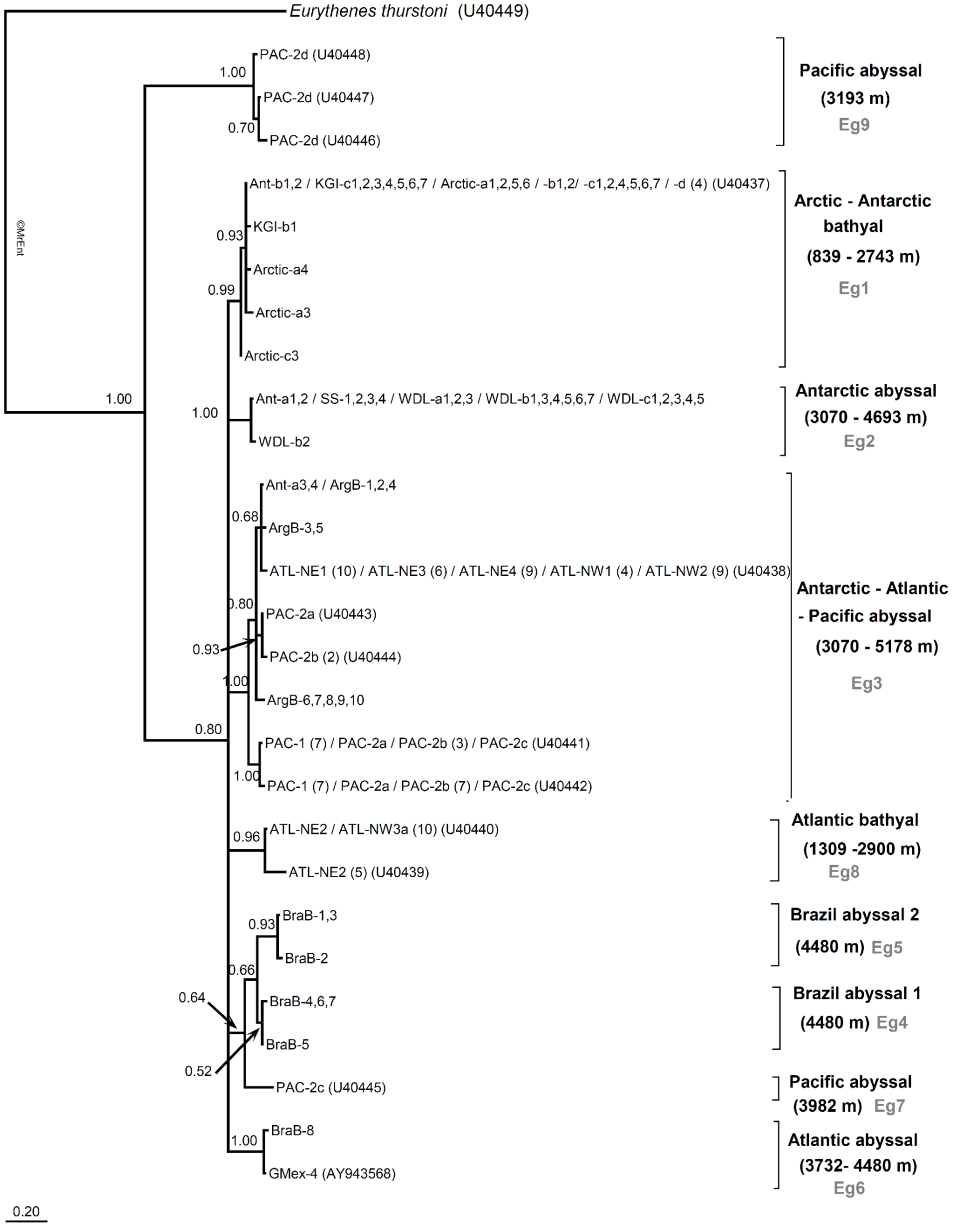
Bayesian tree inferred for the 16S rDNA dataset of *Eurythenes gryllus*. Posterior probabilities (>0.5) are shown at each node. In the case of identical sequences, all specimens are listed with corresponding abbreviations. For the sequences retrieved from GenBank [12], [36], the accession number of the haplotype as well as the number of specimens per haplotype is indicated (when higher than 1). The different clusters are assigned with the codes Eg1–9. For each cluster, distributional ranges (ocean basin, bathyal vs. abyssal, depth) are indicated.

The 16S dataset consisted of 163 sequences representing 27 haplotypes. In the Bayesian tree (Fig. 3), the same clades (Eg1–5) were recovered as with the combined dataset, complemented by four additional clusters (Eg6–9), of which all were supported by posterior probabilities higher than 0.95, except Eg4. The cluster comprising specimens from the Antarctic and Atlantic (Argentine) abyssal sites (Eg3) also included specimens from several abyssal sites in the North-Atlantic and the Pacific Ocean. In addition to the clades Eg4 and Eg5, comprising specimens from the Brazil abyssal Basin, another abyssal cluster (Eg6) was revealed comprising a specimen from the Brazil Basin (BraB-8) and a specimen from the Gulf of Mexico (GMex-4). Additional clusters grouped (Eg8) specimens from bathyal locations in the Iceland Basin (ATL-NE2) and the Bahamas (ATL-NW3) and (Eg9) abyssal specimens from the slope of the Pacific seamount Horizon Guyot. Moreover, a single sequence (Eg7) of a specimen from the same seamount (PAC-2c) appeared to be divergent from all other clusters. Each cluster exclusively grouped specimens from either bathyal or abyssal sampling localities.

**Figure 4:**
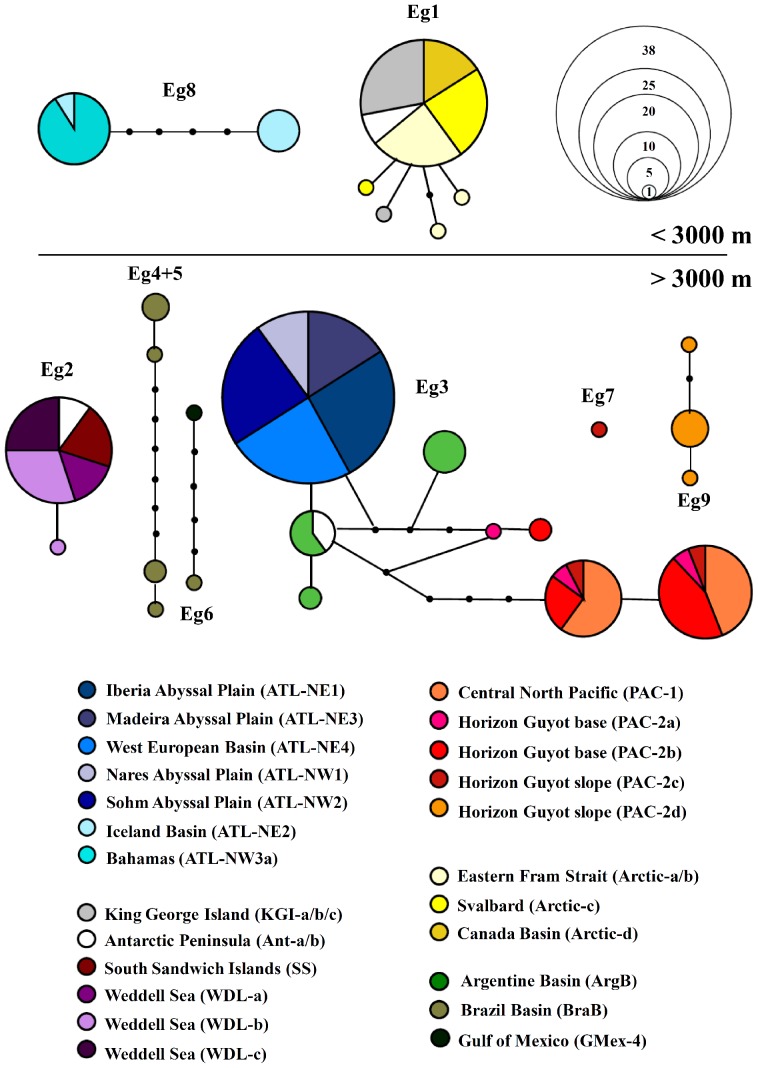
Statistical parsimony haplotype networks based on the 16S rDNA sequences of *Eurythenes gryllus*. The dataset includes sequences from this study, that of France and Kocher [12] and Escobar-Briones *et al.* [36]. The area of each circle is proportional to the frequency of the haplotype in our sampling (a scale is presented). Each line represents a single substitution, nodes represent hypothetical haplotypes and colors refer to the sampling localities. Haplotype networks (95% probability threshold) are numbered (Eg1–9) according to the different clusters identified in Figures 2–3.

The results of the statistical parsimony network analysis on the 16S dataset (Fig. 4) recovered the clusters identified above as unconnected haplotype networks, except for Eg4 and Eg5, which were grouped in one network. Eg7 represented an isolated singleton. Networks showed no overlap between bathyal and abyssal depths: two networks grouped specimens exclusively from above 3,000 m depth each and six networks comprised only specimens from below 3,000 m. Out of 27 different haplotypes, only one was shared between oceans, consisting of bathyal specimens from the Arctic and Southern Ocean (Eg1). Haplotypes were shared between basins in: Eg8, between the Iceland Basin and the trench ‘Tongue of the Ocean’ (Bahamas); Eg2, between the Antarctic Peninsula, Scotia and Weddell seas; Eg3, between the Iberia, Madeira, Nares and Sohm abyssal plains and the West European Basin; Eg3, between the Antarctic Peninsula and the Argentine Basin and Eg3, between the Central North Pacific and the Horizon Guyot seamount.

The level of COI sequence divergence between the five clades (Eg1–5) was high: K2P distances ranged from 4.5 to 13.2% (Table 2). Furthermore, a clear barcoding gap between the highest intraclade (2.0%) and lowest interclade (4.5%) divergences could be observed. Distances within the ‘bipolar’ clade (Eg1) of Antarctic and Arctic bathyal specimens, ranged from 0.0 to 2.0%. The lowest value of interclade divergence (4.5%) represents the distances between specimens from Eg4 and Eg5. When considering Eg4 and Eg5 as a single clade comprising all Brazil abyssal specimens, this value reaches 9.4%. In this case, all clades satisfy the ‘4×’ criterion [57], which assumes that clusters are independent species-level lineages when all pairwise divergences between clusters exceed four times the maximum divergences within the clusters. For COI and 16S, mean interclade divergences (ranging from 9.7 to 11.0% and from 3.5 to 9.0%, respectively) were at least one order of magnitude higher than mean intraclade divergences (ranging from 0.0 to 0.7% and from 0.0 to 0.8%, respectively). For all three genes, mean interclade divergence exceeded at least four times mean intraclade divergence for each clade. However, for 16S and 28S, an overlap between the highest intraclade (i.e. 0.1% and 1.6%, respectively) and the lowest interclade divergences (i.e. 0.1% and 1.4%, respectively) could be detected (Table 2). Since the lowest interclade divergences correspond to the ones between specimens of Eg4 and Eg5, this overlap disappears when considering these as a single species-level clade. This would increase the lowest interclade divergence to 0.3% for 28S and 3.0% for 16S. The clade Eg3 showed a comparatively higher intraclade divergence for 16S (1.6%) than for COI and 28S. This is caused by the inclusion in the 16S dataset of the more divergent sequences of specimens from the Pacific Ocean (PAC-1/2a/2b/2c), which were not available for sequencing of the other genes.

**Table 2:** Range and mean of pairwise K2P intraclade and interclade distances for COI, 28S rDNA and 16S rDNA for each clade identified within *Eurythenes gryllus* (sequence data from this study, France and Kocher [12], Escobar-Briones *et al.* [36]).

	Intraclade (K2P) divergences		Interclade (K2P) divergences	
	Min. – Max.	Mean	Min. – Max.	Mean
**COI**				
Antarctic - Arctic bathyal clade (Eg1)	0.0–0.02	0.007	0.085–0.132	0.101
Antarctic abyssal clade (Eg2)	0.0–0.004	0.001	0.085–0.129	0.097
Antarctic - Atlantic abyssal clade (Eg3)	0.0–0.013	0.006	0.090–0.119	0.104
Brazil abyssal clade 1 (Eg4)	0.0–0.0	0.0	0.045–0.115	0.102
Brazil abyssal clade 2 (Eg5)	0.0–0.003	0.002	0.045–0.132	0.110
**28S rDNA**				
Antarctic - Arctic bathyal clade (Eg1)	0.0–0.001	0.0	0.003–0.007	0.005
Antarctic abyssal clade (Eg2)	0.0–0.0	0.0	0.002–0.006	0.004
Antarctic - Atlantic abyssal clade (Eg3)	0.0–0.001	0.001	0.004–0.007	0.006
Brazil abyssal clade 1 (Eg4)	0.0–0.0	0.0	0.001–0.004	0.003
Brazil abyssal clade 2 (Eg5)	0.0–0.0	0.0	0.001–0.004	0.003
**16S rDNA**				
Antarctic - Arctic bathyal clade (Eg1)	0.0–0.006	0.001	0.022–0.097	0.035
Antarctic abyssal clade (Eg2)	0.0–0.002	0.0	0.022–0.092	0.038
Antarctic - Atlantic - Pacific abyssal clade (Eg3)	0.0–0.016	0.008	0.024–0.102	0.040
Brazil abyssal clade 1 (Eg4)	0.0–0.002	0.001	0.014–0.090	0.038
Brazil abyssal clade 2 (Eg5)	0.0–0.002	0.001	0.014–0.089	0.048
Atlantic abyssal clade (Eg6)	0.002	/	0.036–0.107	0.044
Pacific abyssal singleton (Eg7)	/	/	0.022–0.092	0.042
Atlantic bathyal clade (Eg8)	0.0–0.009	0.004	0.029–0.092	0.041
Pacific abyssal clade (Eg9)	0.0–0.007	0.002	0.078–0.107	0.090

Ultrametric trees obtained for 16S sequences were subjected to the GMYC analysis. The number of ML clusters ranged from eighteen (single threshold method) to thirty-two (multiple threshold method) (Table 3), which did not correspond to the number of clusters recognized in the trees. This can be explained by the limited taxon sampling [56]. Indeed, the full potential of this method could not be used because the large number of species-level clades led to an underrepresentation of intraspecific patterns in our data. However, for each test, the likelihood of the null model (that all specimens belong to a single species) was significantly lower than the maximum likelihood of the GMYC hypothesis (several species-level lineages).

**Table 3:** Results of the GMYC species delimitation test for *Eurythenes gryllus sensu lato* on the 16S dataset using single and multiple threshold methods.

Dataset	Method	Likelihood ofnull model	Maximum likelihood ofGMYC model	Ratio	P value	Number of MLclusters	Confidenceinterval	Thresholdtime(s)
16S	single	31.9843	38.171	12.37341	0.006**	18	3–24	-7
16S	multiple	31.9843	51.79457	39.62055	5.2×10^−08^***	32	32–32	-5 -1

Statistical probabilities **P<0.01 and ***P<<0.0001.

### Morphological Analyses

**Figure 5:**
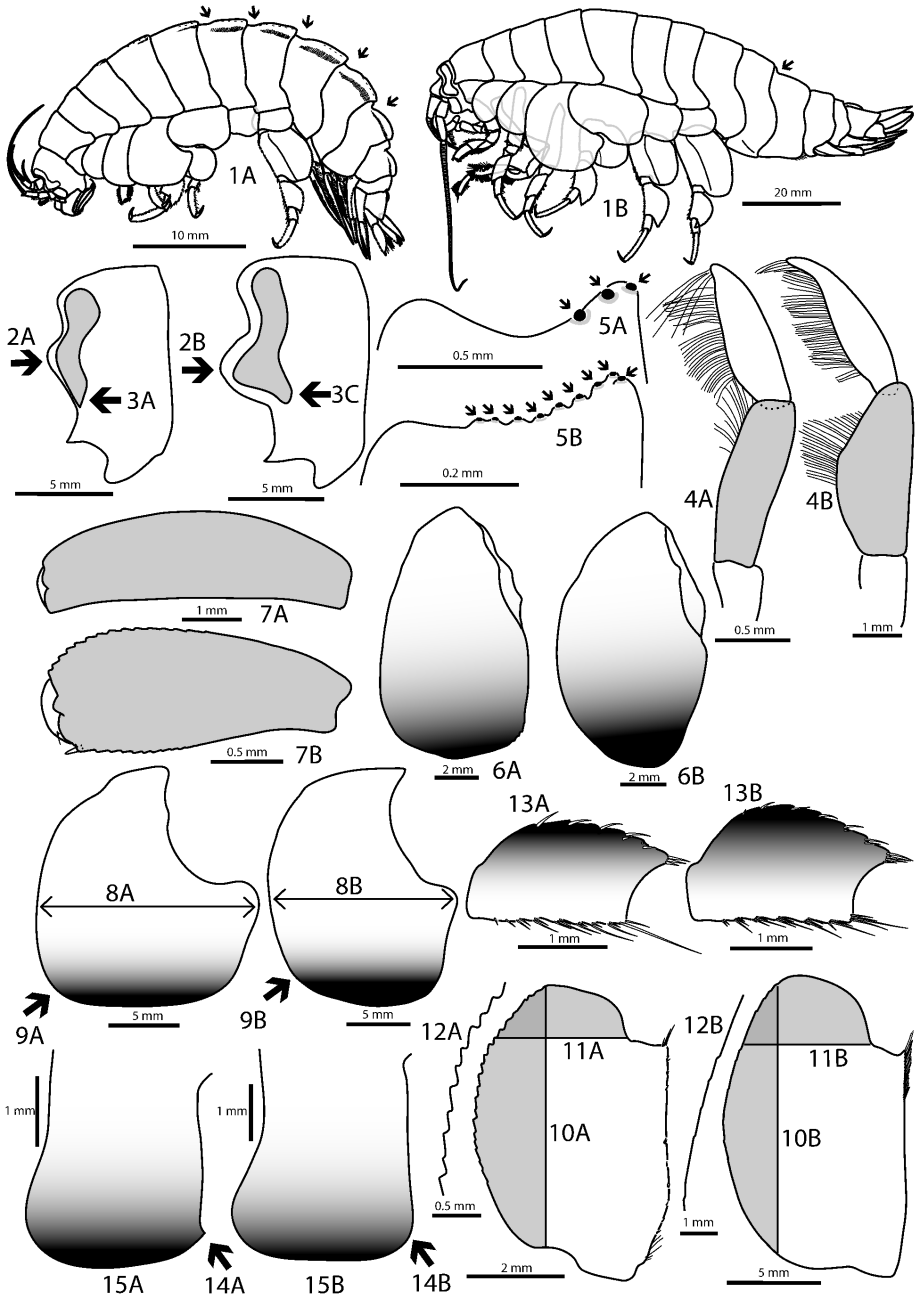
Major morphological differences observed between *Eurythenes* clades. Numbers (1–15) refer to the numbers of the characters presented in Table 4. Illustrated specimens have either been sequenced (*) or belong to genetically and morphologically homogeneous samples, of which some specimens have been sequenced and the best preserved specimens have been illustrated (**). (1) A, specimen BraB-8* from Eg6; B, specimen ArgB-7* from Eg3, (2)(3) A, specimen KGI** from Eg1; B–C, specimen ArgB-7* from Eg3, (4) A, specimen BraB-8* from Eg6; B, specimen ArgB-7* from Eg3, (5) A, specimen KGI** from Eg1; B, specimen ArgB-7* from Eg3, (6) A, specimen KGI** from Eg1; B, specimen ArgB-7* from Eg3, (7) A, specimen KGI** from Eg1; B, specimen ArgB-7* from Eg3, (8)(9) A, specimen KGI** from Eg1; B, specimen ArgB-7* from Eg3, (10)(11)(12) A, specimen BraB-8* from Eg6; B, specimen ArgB-7* from Eg3, (13) A, specimen Ant-a1* (juvenile) from Eg2; B, specimen ArgB-4* (juvenile) from Eg3, (14)(15) A, specimen Ant-a1* (juvenile) from Eg2; B, specimen ArgB-4* (juvenile) from Eg3.

A careful examination of the specimens sampled in this study revealed small but consistent phenotypical differences between Eg1, Eg2, Eg3, Eg4+5 and the genetically divergent specimen of the Brazil abyssal Basin (BraB-8) from Eg6. For Eg1, adult specimens of both sexes were available for examination; for Eg2 only juveniles (max. size 35 mm); for Eg3, one adult male and a few juveniles (see Table 4). For Eg4+5 and Eg6, the specimens examined were medium-sized and it was not clear if they represent adults or not. The genus *Eurythenes* is known to exhibit almost no sexual dimorphism (e.g. [21]). Allometric differences were observed within each clade examined in this study, such as a reduction in length of spines with increasing body size. For the specimen(s) of each clade, a unique combination of character states could be identified. These character states are presented in Table 4 and the most striking interclade differences are illustrated in Figure 5. Clades Eg1, Eg2, Eg3 and Eg6 each exhibit unique and clear-cut character states. In specimens of Eg1, the lower part of the eye is pointed and ventrally directed, whilst it is blunt and pointing obliquely downwards in specimens of other clades. Coxa 2 is ventrally narrowly elliptic in specimens of Eg3, whilst it is broadly rounded in specimens belonging to other clades. Pereionites 6–7 and pleosomites 1–3 are characterized by sigmoid dorsal crests for specimens of Eg6. Specimens of other clades do not bear any such crests and only pleonite 3 has a sigmoid profile. Specimens of Eg2 are characterized by a narrower merus of pereiopod 7 compared with specimens from other clades. Although no unique distinguishing character states were detected for specimens belonging to Eg4+5, they are combined in an arrangement distinct from other species (see Table 4). No differences were detected between the clades Eg4 and Eg5, although characterized by relatively high genetic divergences. Overall, we recognized five putative morphospecies within *E. gryllus* based on the material examined, of which extensive descriptions will be presented elsewhere.

**Table 4:** Summary of the morphological differences observed between specimens from the different clades detected within *Eurythenes gryllus*.

	Eg1	Eg2	Eg3	Eg4+5	Eg6
Samples investigated	KGI-a/b/c; Ant-b; Arctic-a/b/c	WDL-a/b/c; Ant-a1;Ant-a2; SS	ArgB; Ant-a3;Ant-a4	BraB-1,2,3,4,5,6,7	BraB-8
Number, sex and size of specimensinvestigated	30+, both sexes, size range:25–100 mm	30+ juveniles, bothsexes, size range:25–35 mm	1 male, 100 mm; 10juveniles, <35 mm	10+, both sexes,sizerange: 40–50 mm	1 female, 50 mm
1. Pereion and pleosome segments with distinct dorsal sigmoid profile	pleonite 3	pleonite 3	pleonite 3	pleonite 3	**pereionites 6+7 and** **pleonites 1–3**
2. Anterior lobe of the head	**weakly produced**	strongly produced	strongly produced	strongly produced	strongly produced
3. Ventral corner of the eye	**acute and pointing** **linearly downwards**	blunt and pointingobliquely downwards	blunt and pointingobliquely downwards	blunt and pointingobliquely downwards	blunt and pointingobliquely downwards
4. Mandible palp: article 2	Narrow	narrow	**broad**	Narrow	narrow
5. Inner plate of maxilliped: anteriornodular spines	3–4, not protruding	3, not protruding	8–9, not protruding	**3–6, protruding**	**9–10, protruding**
6. Coxal plate 2: ventral edge	weakly curved	weakly curved	**strongly curved**	weakly curved	weakly curved
7. Propodus of gnathopod 2, profile	long, narrow	fairly short, fairlynarrow	long, narrow	short, broad	**very short, very** **broad**
8. Coxal plate 4: width	**Broad**	fairly broad	**narrow**	Variable	fairly narrow
9. Coxal plate 4: anteroventral angle	bluntly angular	bluntly angular	**rounded**	bluntly angular	bluntly angular
10. Basis of pereiopod 7: posterior lobe	strongly expanded	strongly expanded	**moderately** **expanded**	strongly expanded	strongly expanded
11. Basis of pereiopod 7: distal lobe	moderately protruding	moderately protruding	**strongly protruding**	moderatelyprotruding	moderately protruding
12. Basis of pereiopod 7: crenulation	medium to strong	strong	**weak to** **very weak**	medium tostrong	strong
13. Merus of pereiopod 7	Broad	**narrow**	broad	broad	broad
14. Epimeral plate 3: tooth (in specimens≤35 mm)	Absent	present	absent	present or absent	absent
15. Epimeral plate 3: profile of ventral margin	strongly curved	strongly curved	**weakly curved to** **nearly straight**	weakly to stronglycurved	weakly curved

Distinguishing character states are indicated in bold.

## Discussion

### Molecular Evidence for Multiple Species Within Eurythenes Gryllus

We identified nine lineages within *E. gryllus* on the basis of the sequences available for all specimens (16S rDNA), five of which were also corroborated by the results of the analyses on the three-gene dataset. Analyses based on mitochondrial markers revealed similar clades as those focusing on nuclear data, showing that the results are not an artifact of gene tree – species tree discordance. For all three genes, every clade but Eg4 and Eg5 satisfied the ‘4×’ criterion [57], which has been successfully applied to mitochondrial data for (cryptic) species delimitation in amphipod crustaceans [58]. Despite the limitations caused by small intraclade sampling, the GMYC species-delimitation tests also supported the existence of multiple species over a single species. Nonetheless, further sampling is needed to confirm that the lineages, which were identified exclusively based on the 16S sequence dataset (e.g. the single sequence Eg7), represent species-level clades.

For COI, a clear barcoding gap could be observed. The level of interclade divergences was found to be in concordance with interspecific divergences reported for lysianassoid amphipods (6.3 to 20.1%, [54]), except for Eg4 and Eg5. For 28S and 16S, there was an overlap between the highest intraclade and the lowest interclade divergences, which disappeared when considering Eg4 and Eg5 as a single clade. Furthermore, several cases of sympatry of genetically distinct clades were observed: (i) abyssal specimens from the Antarctic Peninsula (Ant-a) appeared both in Eg2 and Eg3, (ii) specimens from the Pacific (PAC-2c) were recovered in cluster Eg3 and as divergent sequence Eg7 and (iii) specimens from the Brazil Basin represented either of three different clades (Eg4, Eg5 and Eg6). The bimodal distribution of intra- and interspecific divergences in combination with the prevalence of species-level differentiation of sympatric specimens are considered as evidence of cryptic or hidden species [59]. Eg4 and Eg5 represent sympatric clades that were well-supported for the three-gene dataset and partly for the 16S dataset. A clear barcoding gap can be observed between intra- and interclade divergences for COI, but not for 16S and 28S. Hence, this might correspond to a case of recent or ongoing speciation.

### Morphological Findings

The current taxonomy of many deep-sea groups is conservative [10], [12]. In several cases, the assignment of a widespread distribution to deep-sea species is believed to be based on misidentifications or overlooking subtle morphological differences [10]. In addition, the evaluation of intraspecific variability is hampered due to limited sampling. A reverse taxonomy approach (e.g. [60]) was applied to the newly collected samples resulting in the recognition of five overlooked morphospecies. However, no morphological differences could be detected between Eg4 and Eg5. Specimens from the bipolar clade (Eg1) were collected in proximity of the type locality (Greenland Sea). Of all specimens examined, those of Eg1 were the most similar to the holotype of *E. gryllus* (illustrated by Stoddart and Lowry [21]). Hence, the specimens from Eg1 are likely to represent the ‘true’ *E. gryllus* even if upon examination of the holotype, the anterior lobe of the head appeared to be slightly longer than that of the specimen of clade Eg1. Nonetheless, this can only be ascertained upon an examination of specimens from all lineages and in particular the specimens belonging to the bathyal North-Atlantic clade (Eg8), for which no specimens were available for examination.

### Geographic and Bathymetric Patterns

The bathyal clade Eg1 comprised specimens from both Arctic and Antarctic regions, sampled from 839 to 2,743 m depth. Recently, the Census of Marine Life counted at least 235 organisms (from whales to small invertebrates) with a bipolar distribution. However, identification of these bipolar species has been exclusively based on morphological characters and might be biased due to morphological convergences or a (perceived) lack of distinctive characters [61]. Often, upon a detailed morphological analysis or molecular studies, bipolar species were shown to be composed of several morphospecies (e.g. [62]) or genetically divergent, cryptic species (e.g. [63]), with a distribution restricted to a single pole. Based on an extensive morphological analysis, a benthic bryozoan species has been identified as a strong candidate for a bipolar distribution, however not yet confirmed by molecular evidence [64]. To our knowledge, genetically similar species in Arctic and Antarctic oceans have only been observed in bacteria and archaebacteria [65], [66] and benthic foraminifera [67]. Hence, this is the first molecular evidence for a bipolar distribution in a macro-benthic deep-sea organism: specimens of *E. gryllus* separated by distances up to 16,750 km were characterized by low genetic divergences (COI: 0.8–2.0%) sharing a single 16S haplotype. Gene flow between Antarctic and Arctic populations might be facilitated by passive transport with the thermohaline circulation, i.e. the Antarctic Bottom Water (AABW), originating in the Weddell Sea and covering much of the world ocean floor [68]. Alternatively, recent colonization events could explain the occurrence of specimens with identical 16S haplotypes across different basins. Specimens might have dispersed repeatedly from one pole to another across the tropics, during the Quaternary glacial cycles, possibly by submergence into deeper waters [69]. Nonetheless, we cannot rule out the hypothesis of a cosmopolitan distribution of Eg1, with populations also occurring at lower latitudes.

Hydrography might also play an important role in connectivity between abyssal populations of clade Eg3 for which identical 16S haplotypes were found (i) throughout the entire North Atlantic Ocean, (ii) in the Argentine Basin and around the Antarctic Peninsula and (iii) at two localities in the Central North Pacific. In this case, the Antarctic Circumpolar Current combined with the northbound movements of the AABW might be responsible for dispersal between these basins. Future population genetic studies based on fast-evolving nuclear markers will allow us to test these hypotheses. Oppositely to hydrographic features, topography might also play a role in population connectivity of deep-sea organisms since undersea mountain chains, rises and continents can act as barriers for dispersal [70]. However, the distribution of *E. gryllus sensu lato* did not seem restricted due to geological features, since genetic homogeneity was observed between abyssal populations in the Argentine Basin and around the Antarctic Peninsula as well as between populations in the northwest and northeast Atlantic, across the American Antarctic Ridge and the Mid-Atlantic Ridge, respectively.

In contrast to these cases of genetic homogeneity observed over enormous geographic distances, high genetic divergences could be detected over small bathymetric distances. Specimens sampled at 3,070 m near the Antarctic Peninsula (Ant-a) were separated from specimens from the same region (Ant-b) but sampled at shallower depths (≤2,743 m) by genetic distances in the range of interspecific divergences. This break around 3,000 m also appeared in the trees and haplotype networks, since there was neither cluster nor network that would unite specimens from both above and below this limit. Accordingly, environmental gradients determining the distribution of *E. gryllus* seem to be associated with depth. It has been shown previously that small bathymetric changes appear much more significant for promoting population differentiation than geographic distance in a variety of animal groups: corals [71], gastropods [72], bivalves [8], [15], polychaetes [73], asteroids [74], fish [75] and eusirid amphipods [76]. This might be explained by the fact that several potentially important selective agents vary with depth, e.g. sediment characteristics [3], composition and rates of nutrient supply [77], the nature and intensity of biotic interactions [78] and a direct effect of hydrostatic pressure on physiological traits [79].

Vertical partitioning of closely related species has also been observed in other deep-sea benthic [33], [80] and pelagic crustaceans [81]. In deep-sea lysianassoids, several examples of differing depth ranges for congeneric species exist, e.g. for *Paralicella* [80]. This is thought to result from competition for resources [27], [81] or to be linked with the occurrence of favorable habitats at particular depths. These suitable habitats could be determined by the physiological limits of a species in relation to the synergistic effects of high pressure and low temperature [82]. For example, the maximum pressure tolerated at a certain temperature by the bathyal lysianassoid *Stephonyx biscayensis*, is consistent with its bathymetric and geographic distribution [83]. For *E. gryllus*, a genetic separation appeared between 2,743 (2,900 in case of the Atlantic bathyal species) and 3,070 m depth. For the studied specimens of *E. gryllus* by France and Kocher [12], this genetic break occurred between 3,200 and 3,500 m; for the bivalve *Deminucula atacellana* between 3,000 and 3,300 m depth [15]. Based on observations for different organisms from different regions, a ubiquitous phylogeographic barrier for barophysical tolerance was assumed to occur around 3,000–3,500 m [9], [15]. Similar to the ecotone (i.e. a narrow transition of a distinct species composition between two habitats) observed for scavenging amphipods between abyssal and hadal depths [84], this genetic break might represent evidence for an ecotone at the transition between bathyal and abyssal depths. Selection might have favored enzymatic adaptations or modified proteins below this transition, thus promoting an effective barrier to exchange between bathyal and abyssal depths. Nevertheless, enzymes and proteins differ in their sensitivity to hydrostatic pressure and species may differ in their responses [79]. In addition, *E. gryllus* can easily tolerate pressure differences since specimens are easily maintained alive for longer periods when retrieved from depths around 2,000 m (C. Held, personal observation). Furthermore, this species is known to accomplish ontogenetic migrations throughout the water column, with adult individuals moving into higher water layers, adopting a pelagic life-style (e.g. [32]). Hence, although *E. gryllus* is able to migrate vertically through water column without ill effects, our results suggest its reproduction to be limited to a more confined depth range.

### The Depth-differentiation Hypothesis Revisited

More genetic lineages were encountered in abyssal than in bathyal regions: two species-level clades could be distinguished above 3,000 m and seven species-level clades below 3,000 m. This finding is in contrast with the depth-differentiation hypothesis, stating that genetic differentiation between populations decreases with depth (reviewed in [9]). This hypothesis also implies that species diversity (e.g. [3]), intraspecific morphological variation and zonation (e.g. [8]) are higher at bathyal depths. Since the upper bathyal zone is topographically more complex and characterized by more heterogeneous sediments than the supposedly uniform abyssal environment [3], the potential for population differentiation as well as co-existence of differentially adapted species may be higher at bathyal depths (e.g. [8]). A greater level of genetic differentiation in the bathyal zone was previously hypothesized for *E. gryllus*, as more clades were observed above 3,200 m, than below, and the abyssal lineage was assumed to be widespread [12]. Our results, based on a more extensive dataset, show the opposite: higher (species) diversity in the abyss. The two bathyal species-level clades identified here are both characterized by a very wide geographic range: one is bipolar and the other is found to occur both in a Bahamian trench and in the Iceland Basin. In the study of France and Kocher [35], the latter was found in a species-level clade together with specimens from the Lau Basin, a relatively shallow basin situated in the southwest Pacific, attributing to both bathyal species-level clades a very wide geographic range. Hence, despite the more uniform environment at abyssal depths, gene flow could be considerably less than in the bathyal zone. However, we cannot exclude biases due to our sampling effort, which was numerically still rather limited, including only a few individuals per locality. Moreover, it should be taken into account that sampling effort for this study was more focused on the abyss, covering more ocean basins at depths below 3,000 m. Hence, a number of haplotypes might not have been sampled, which has an implication on the assessment of genetic diversity.

## Conclusions

Although *Eurythenes gryllus* is the most intensively studied taxon of all deep-sea amphipods throughout the world ocean, genetic and morphological differences are still overlooked. Genetic evidence suggests that *E. gryllus* might represent at least nine species-level lineages, with distinct, partly overlapping, geographical ranges. However, bathymetric distributions seem to be confined to depths either above or below 3,000 m. Two bathyal and two abyssal species-level clades showed a widespread distribution, while five other abyssal species-level clades seemed restricted to a single ocean region. This challenges the general assumption that the abyss has a more limited potential for speciation events to occur due to its homogeneous environment and lack of obvious isolating barriers. If such unexpectedly high species diversity is observed for a giant and highly mobile amphipod, we predict the abyss to be much more complex and more likely to harbor an important hidden invertebrate diversity than previously assumed. Molecular studies supporting the depth-differentiation hypothesis are scarce and often based on a limited sampling. Extending the sampling coverage gave contrasting results to previous assumptions. These biases due to sampling effort clearly highlight the difficult nature of deep-sea research. In addition, deep-sea organisms are known to be patchily distributed (e.g. [85]), which increases the need of additional sampling to fully evaluate species distributions. Finally, our results show that the deep sea still is a widely unexplored realm and further studies, particularly of environments such as seamounts and trenches, are likely to shed light on an even higher number of species.
